# Nanoparticles in Bioimaging

**DOI:** 10.3390/nano6060105

**Published:** 2016-06-06

**Authors:** Yurii K. Gun’ko

**Affiliations:** 1 School of Chemistry and CRANN, Trinity College Dublin, Dublin 2, Ireland; 2 ITMO University, St. Petersburg 197101, Russia

This Special Issue of *Nanomaterials* is dedicated to the application of nanoparticulate materials in biological imaging. The issue highlights the rapidly expanding uses and interest in nanomaterials for bioapplications; in particular, bioimaging. There are three reviews and five experimental research papers in this issue, focused around this area. Some highlights of the publications in the issue are discussed below.

In their review article, Zhang *et al.* [1] present current achievements in the development of new dye-loaded fluorescent silica nanoparticles (SiNPs) for non-invasive fluorescence bioimaging; both *in vitro* and *in vivo*. SiNPs are easy to functionalise and demonstrate good biocompatibility, low toxicity, high hydrophilicity and excellent optical transparency. Due to these properties, SiNPs are suitable substrates for the fabrication of fluorescent probes used in the effective imaging of living cells and small animals. The authors also discuss a number of challenges that limit applications of these nanomaterials, such as leakage of dyes from the fluorescent SiNPs, nonspecific binding and their dispersibility in aqueous media.

In another review article [2], the authors discuss recent advances in cancer tumor imaging and thermal therapy, using multifunctional inorganic nanoparticles. There is a broad range of nanomaterials addressed in this article, including metal (Au, Ag, Pt, Pd), magnetic (e.g., iron oxides), NIR-absorbing carbon (e.g., graphene and carbon nanotubes) and quantum dot (e.g., CdTe, CdSe) based nanostructures, and upconversion composite nanoparticles (e.g., NaGdF_4_:Yb:Er). Particular attention was paid to the development of nanoparticles as theranostic tools and the dual application of nanomaterials for both thermal therapy and biological imaging.

The third review article [3] is dedicated to lanthanide-doped upconverting nanoparticles (UCNPs) and their use in bioimaging. These nanomaterials can convert low energy near-infrared (NIR) photons into high energy UV or visible emission using multiphoton upconversion processes. The authors describe engineering of UCNPs for biomedical applications, their various uses as imaging contrast reagents, imaging guidable drug delivery nanoplatforms, and phototherapeutic reagents. However, despite the significant recent progress in this field, there are still a number challenges, such as low upconversion efficiency of lanthanide-based UCNPs, difficulties with producing small sub-10 nm UCNPs, and development of more versatile, commercially available UCNP systems. These challenges are to be addressed in the near future.

In their experimental work, Ahlinder *et al.* [4] investigated the biodistribution of polystyrene nanoparticles in A549 lung epithelial cells using confocal Raman spectroscopy imaging. Algorithms were also applied for image enhancement. The use of Tikhonov regularization enabled the authors to reduce noise in images of score maps. The super-resolution-treated images have shown particle aggregates inside cells. The images have been compared by the evaluation of the intensity profiles of line mappings over particle agglomerates visible in the images and comparison with threshold images. It was found that the resolution after application of super-resolution algorithms is not significantly improved compared to the theoretical limit for optical microscopy. Nevertheless, these methods help to reduce noise and artefacts in the hyperspectral Raman images while maintaining a good spatial resolution. The authors have proposed the use of this methodology for Raman imaging of various biological samples and other photo-sensitive samples.

Another experimental paper presents an investigation of automatic echographic detection of halloysite clay nanotubes (HNTs) as potential contrast agents for ultrasound targeted imaging at a conventional diagnostic frequency of 10 MHz. [5]. In these studies characteristic “signatures” of HNTs in the radio-frequency backscatter signals were used to develop tailored colour maps. These were then superimposed on conventional B-mode echographic images for automatic HNT detection with the sensitivity up to 60% and specificity above 95% at the HNT concentration of 1.5 mg/mL. The clinical implementation of this technique and specifically functionalized HNTs, should potentially result in significant diagnostic improvements enabling non-ionizing identification of pathological tissues at cellular level.

Xia and Wen [6] report here the synthesis of nickel nanowires with tunable characteristics and a range of potential applications. Due to the anisotropy and high aspect ratio, the nickel nanowires exhibited an enhanced saturation magnetization over bulk nickel metal. It was found that the size and morphology of the nanowires can be adjusted by tuning the reaction temperature, time, and the surfactant concentration. The authors believe that this technique could be further developed for an industrial scale-up fabrication of nickel nanowires. 

An interesting experimental work on selective labeling of proteins on living cell membranes using fluorescent nanodiamond based assays is presented here by Tochio and co-workers [7]. The researchers used hyperbranched polyglycerol to modify fluorescent nanodiamonds, which then were conjugated to proteins expressed on the plasma membrane *via* mutated β-lactamase-tag. This approach has enabled site-specific labeling of the Interleukin-18 receptor alpha (IL18Rα) chain expressed on the plasma membrane of human embryonic kidney 293 (HEK293) cells. Single-molecule tracking techniques allowed the researchers to perform imaging of the diffusion trajectory of fluorescent nanodiamond labeled IL-18Rα on the cells. It is expected that these nanomaterials and approaches should open up new opportunities for cellular bioimaging of proteins and other nanobiotechological applications.

Cui *et al.* reported the synthesis and characterization of Gd-functionalized gold nanoclusters for potential applications as multimodal fluorescent/MRI/CT Contrast Agents [8]. These multimodal nanomaterials have been prepared by biomineralisation using bovine serum albumin (BSA) as a mediator and stabilizer. It was found that the new Gd-Au nanoclusters exhibit fluorescent properties and a high longitudinal relaxivity value of 22.111 s^−1^ per mM of Gd in phosphate-buffered saline, which is six times higher than that of the commercial MRI agent Magnevist. In addition, these nanomaterials can be used as computed tomography (CT) contrast agents due to the presence of Au element, as it was confirmed by single photon emission computed tomography (SPECT) and CT studies. Finally, *in vitro* cytotoxicity tests have shown that the Gd-Au nanoclusters have a very low toxicity and good biocompatibility. This research has demonstrated that Gd-Au nanoclusters are very promising multimodal imaging contrast agents.

Overall, the papers in this Special Issue offer new insight into interesting developments in the field of biological imaging using various nanomaterials. We recommend to read through this Special Issue and we look forward to seeing further progress in research on nanomaterials for bioimaging and other related applications.
